# Use of Ultrasound in the Diagnosis of Travel-Acquired Cutaneous Myiasis in a Pediatric Patient

**DOI:** 10.7759/cureus.83640

**Published:** 2025-05-07

**Authors:** Ziad Letaïef, Marie-Sofie Walgraeve

**Affiliations:** 1 Radiology, Universitair Ziekenhuis Brussel, Jette, BEL; 2 Radiology, Algemeen Ziekenhuis Sint-Jan Brugge, Brugge, BEL

**Keywords:** bot fly, myiasis, pediatric ultrasound, skin infections, tropical environment

## Abstract

A pediatric patient presented to the emergency department with ambiguous scalp lesions following recent travel to a tropical region. Despite a prompt physical examination and subsequent laboratory investigations, a definitive diagnosis could not be established. An experienced ultrasound radiologist from our team was consulted to assist in the diagnostic process using ultrasound imaging. The ultrasound revealed the presence of subcutaneous larvae, confirming a diagnosis of cutaneous myiasis, which had initially mimicked a more common dermatological condition. This case highlights the valuable role that ultrasound can play in elucidating rare skin lesions, particularly in regions where clinicians may lack experience with tropical diseases due to their infrequent presentation. Early use of ultrasound in similar cases may expedite diagnosis and treatment, improving patient outcomes and reducing unnecessary interventions.

## Introduction

Bot flies are the most common cause of cutaneous myiasis in humans. The term “bot fly” refers to the members of the Oestridae family [1]. Infestations are extremely rare in Europe and Northern America and occur almost exclusively in tropical travelers. The leading cause of cutaneous myiasis in humans is Dermatobia hominis, a dipterous fly native to Central and South America [2]. The diagnosis of bot fly myiasis is primarily based on clinical examination; however, it may prove challenging due to the rarity of the condition in temperate regions. This report presents a case of an 11-year-old male patient who acquired a bot fly infection during a family vacation in Costa Rica. This case underscores the pivotal role of ultrasound in establishing a diagnosis in rare and perplexing cutaneous conditions such as cutaneous myiasis, particularly when clinical evaluation alone fails to provide clear diagnostic indicators.

## Case presentation

An 11-year-old male patient was referred to the emergency department by his general practitioner with a suspected Staphylococcus aureus infection. He presented with four boil-like lesions on the scalp along with bilaterally swollen retro-auricular and posterior cervical lymph nodes. The general practitioner had initially prescribed empirical antibiotic therapy with amoxicillin for six days, which was subsequently changed to amoxicillin and clavulanic acid due to therapeutic unresponsiveness. Upon further evaluation, the patient's history revealed recent travel to Costa Rica. Dermatological examination identified four pustular lesions on the scalp, each with a central punctum and purulent discharge. The patient reported no additional symptoms. Laboratory investigations showed no signs of inflammation, and a skin swab test for methicillin-resistant Staphylococcus aureus (MRSA) was negative.

Due to the absence of any definitive diagnostic findings, the patient was referred to the radiology department for further evaluation of the scalp lesions and cervical lymph nodes using ultrasound. Ultrasound imaging revealed hyperechoic mobile structures (Video 1) of variable sizes (8-12 mm) within the lesions.

**Video 1:** Ultrasound imaging revealing a hyperechoic mobile structure in one of the scalp lesions

**Figure 1:**
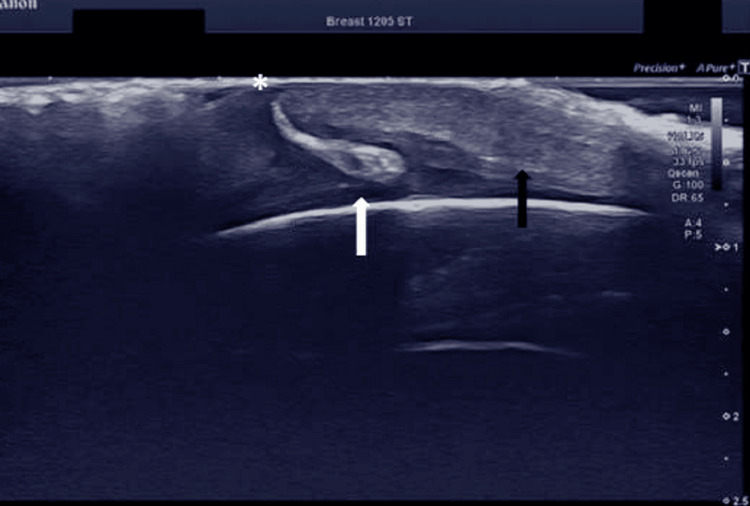
Ultrasound image showing a hyperechoic larval structure (white arrow) embedded in the skin of scalp, surrounded by subcutaneous swelling (black arrow) and a visible entry point (asterisk) at the surface

**Figure 2:**
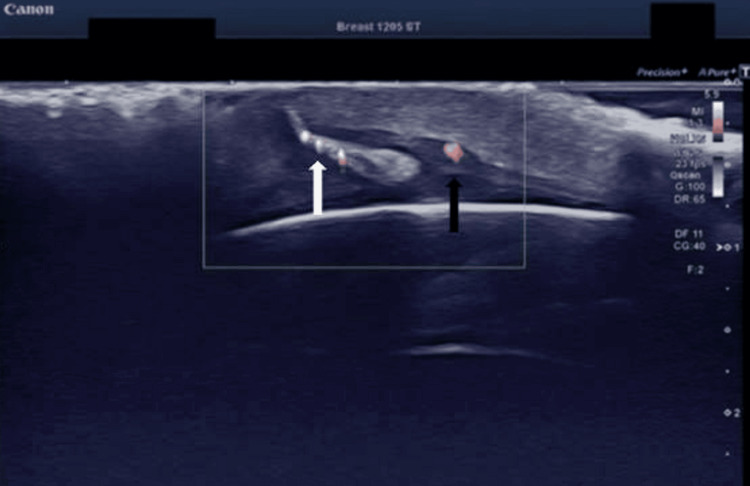
Color Doppler image reveals the vascularity of the larval structure (white arrow) and the accompanying hyperemia of the subcutaneous tissue (black arrow) nearby

Each was associated with a visible entry point and was accompanied by surrounding hyperemia and subcutaneous tissue swelling (Figures 1, 2). 

**Figure 3:**
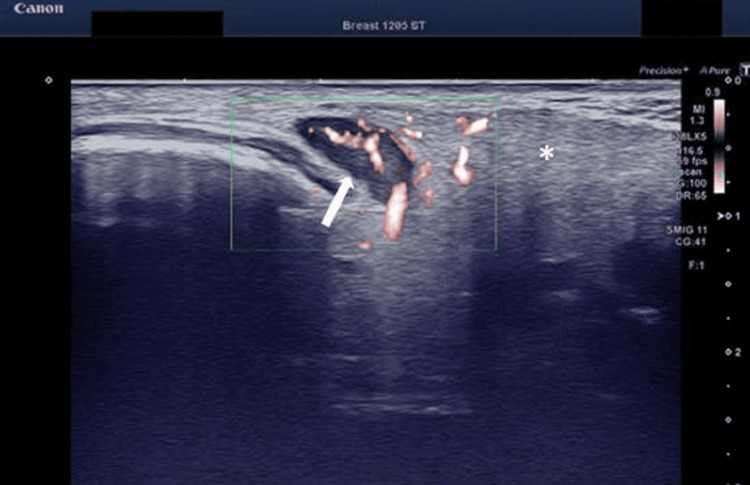
Color Doppler image demonstrates a swollen lymph node (white arrow) with hyperemia and infiltration (asterisk) of the surrounding soft tissue in the posterior cervical region, indicative of lymphadenopathy

Additionally, cervical lymphadenopathy was observed (Figure 3).

**Figure 4:**
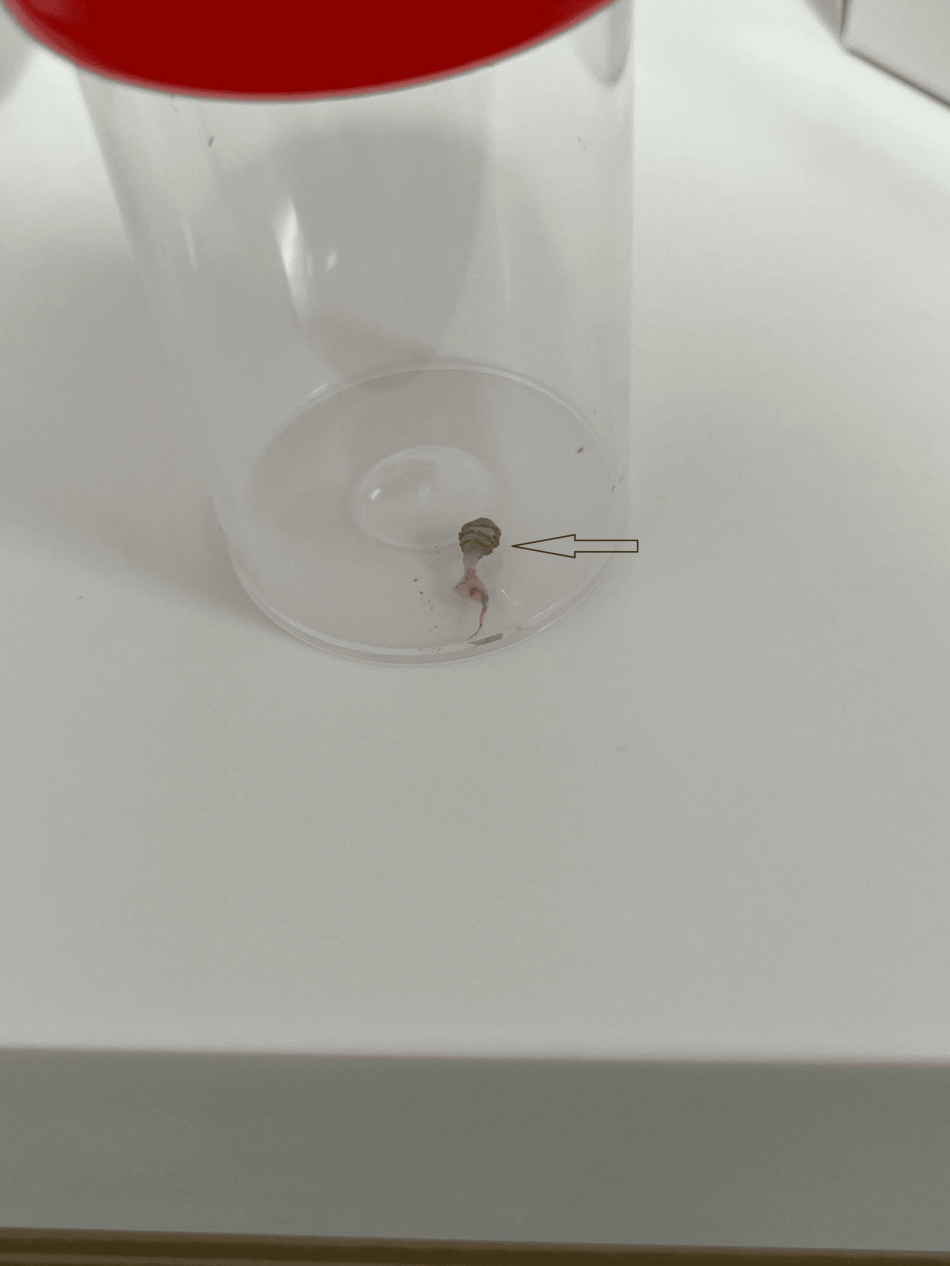
One of the surgically-removed larvae with black spiny circular projections (arrow) The larva displays the characteristic bottle-neck shape of the second instar stage, with small black spiny circular projections present anteriorly, indicative of Dermatobia hominis.

The larvae were surgically removed under local anesthesia (Figure 4).

No entomological analysis was performed to confirm the species. However, based on the clinical presentation, recent travel history to an endemic region, and the characteristic findings on ultrasound, a diagnosis of Dermatobia hominis infestation was made.

## Discussion

Despite the absence of an entomological analysis in this case, the report discusses Dermatobia hominis, the human bot fly, which is the primary cause of furuncular myiasis and is endemic throughout Central and South America. The reproductive cycle of the female bot fly involves depositing up to 30 eggs on the body of hematophagous arthropods, typically mosquitoes, which serve as phoretic vectors. Upon contact with a warm-blooded host, the increase in temperature triggers the hatching of the eggs, allowing the larvae to penetrate through hair follicles or small breaches created by the vector. The larvae develop within the host’s skin over a period of four to 18 weeks before eventually exiting to pupate on the ground. Clinically, the patient develops furuncle-like lesions with a central pore, accompanied by symptoms such as itching and pain [3,4].

The primary treatment is the surgical removal of the larvae under local anesthesia [5]. Although the infestation is usually self-limiting, incomplete removal can lead to a secondary bacterial infection [6]. Therefore, familiarity with this condition is essential for both first-line healthcare providers and radiologists to prevent misdiagnosis and inappropriate treatment when assessing furuncular skin lesions, especially in travelers returning from endemic areas. Radiologic evaluation, particularly through ultrasound, can aid in visualizing larval structures and may serve as the only diagnostic clue, as demonstrated in this case. Ultrasound typically reveals hyperechogenic mobile structures, confirming the diagnosis and guiding appropriate treatment [7,8].

Epidemiological data on the incidence of human bot fly skin lesions in temperate regions is sparse, largely due to the rarity of the condition. Most cases are believed to be imported, and as of the writing of this case report, no official data are available [9]. Increased awareness among healthcare providers, including radiologists, is essential for accurate diagnosis and appropriate management of this rare condition in the temperate regions.

## Conclusions

Several case reports in the literature highlight the diagnostic challenges of cutaneous myiasis, emphasizing the role of ultrasound in evaluating suspicious skin lesions, especially in patients with a history of travel to endemic regions. Ultrasound has proven to be a valuable, non-invasive tool for detecting larvae and differentiating myiasis from other skin conditions, such as furuncles, that may not respond to standard treatments.

To our knowledge, no case report has been published about a pediatric patient presenting with this entity and diagnosed by ultrasound. As such, this case further underscores the importance of considering cutaneous myiasis in the differential diagnosis of boil-like lesions in pediatric travelers to endemic regions, while also demonstrating the diagnostic value of ultrasound when clinical examination alone is inconclusive.

This case also demonstrates that skin lesions in general can be challenging when characterized by unusual or atypical features. Medical imaging, especially by ultrasound, provides a non-invasive and accessible diagnostic tool for dermatological entities and could be widely implemented in daily clinical practice.
